# Immunity to Influenza is dependent on MHC II polymorphism: study with 2 HLA transgenic strains

**DOI:** 10.1038/s41598-019-55503-1

**Published:** 2019-12-13

**Authors:** David Luckey, Eric A. Weaver, Douglas G. Osborne, Daniel D. Billadeau, Veena Taneja

**Affiliations:** 1 Department of Immunology Mayo Clinic, Rochester, MN USA; 2 Present Address: School of Biological Sciences, Nebraska Center for Virology, University of Nebraska, Lincoln, NE USA; 3 Present Address: Department of Dermatology, University of Colorado, Aurora, CO USA

**Keywords:** Influenza virus, Influenza virus

## Abstract

Major histocompatibility complex II (MHC II) molecules are involved in antigen presentation and the development of a functional adaptive immune response. Evolutionary selection for MHC molecules that effectively clear infectious agents provides an advantage to humans. However, certain class II molecules are associated with autoimmune diseases. In this study we infected autoimmune-susceptible DRB1*0401.AEo and non-susceptible *0402.AEo mice with H1N1 influenza and determined clearance and protective immunity to H3N2 virus. *0401 mice generated a robust TLR-triggered immune response and cleared H1N1 influenza virus infection. After vaccination and challenge with H1N1, *0401 mice, when challenged with H3N2, generated cross-protective immunity to heterosubtypic H3N2 influenza strain whereas *0402 mice cleared the H1N1 infection but did not generate cross-protective immunity against the H3N2 influenza strain. The intracellular trafficking route of MHCII revealed that *0401 molecules traffic through the late endosome/lysosomes while *0402 molecules traffic into early endosomes. This suggested that trafficking of MHCII could affect the functional output of the innate immune response and clearance of viral infections. Also, DRB1*0401 mice live longer than HLA-DRB1*0402 mice. The study provides a potential hypothesis for evolutionary selection of *0401 molecule, even though it is associated with autoreactivity, which may be dependent on the availability of peptide repertoire of self-antigens.

## Introduction

Major histocompatibility complex (MHC) class II molecules present peptides to T cells leading to generation of immune responses to pathogens. Classically, MHC II molecules present peptides-derived from extracellular pathogens via trafficking through endosomes, alternatively cytosolic antigens generated via autophagy have been shown to be presented^1–3^. Infectious etiology for autoimmunity has been suggested for many autoimmune diseases including rheumatoid arthritis (RA) though no pathogens have been implicated. Predisposition to RA has been associated with the presence of HLA-DRB1*0401 in most populations^4^. On the other hand, MHC II alleles that share the third hypervariable sequence with *0402 have been associated with a resistance to developing RA. The basic paradigm for the evolutionary selection of the disease susceptible HLA alleles is that these alleles can clear infections efficiently. This is supported by the observations showing that HLA-DR4 was significantly associated with the clearance of hepatitis B virus infection^5^, even though DR4 is associated with susceptibility to RA. DR4 individuals generate a much higher response to epitopes of influenza virus compared to those carrying other MHC II alleles^2^. Recently, 2 studies showed that endogenous processing of influenza virus is required to generate primary CD4 response, suggesting a role of class II molecules^3,6^.

To define the role of MHC II genes, we have generated transgenic mice expressing human HLA class II genes associated with autoimmune diseases but lacking endogenous class II molecules. The effector arm of the immune response is restricted by the HLA genes making relevant to human pathology^7^. This is supported by our previous work with transgenic mice expressing HLA-DRB1*0401 genes, which develop inflammatory arthritis that mimics human RA in disease histopathology, autoantibodies and sex-bias^8^ while DRB1*0402 expressing mice are protected from arthritis^9^. HLA transgenic mice mimic humans in the expression of class II molecules and can present epitopes of a protein similar to that in humans^7^. Using transgenic mice, we showed that the T cells selected by the HLA transgene are predetermined to develop a certain cytokine profile^10^. Thus while certain MHC II alleles can generate robust responses and clear infection much more efficiently than other alleles^2,5^, the ensuing immune response or epitope mimicry may lead to predisposition to disease in genetically susceptible individuals.

The MHC genes also play a major role in age-related response to infections, as most of the diseases during aging have an immunological pathogenesis associated with the decline of T cell responses and increased propensity to autoimmune reactivity. Class II molecules have also been suggested to be involved in clearance of influenza. We have hypothesized that autoimmunity could be the result of selection of MHC molecules, during evolution, MHCII molecules providing protection from infections, which could be dependent on the intracellular endosomal trafficking routes of different MHCII molecules, were selected. In fact, we have previously shown that perturbation of the endosomal trafficking by depletion of either the evolutionarily conserved retromer or WASH (Wiskott-Aldrich syndrome homologue) complexes impacts the loading of antigen on MHC class II molecules leading to impaired T cell activation^11^. In this study, we used 2 strains of mice expressing autoimmune-associated, *0401, or non-associated, *0402, HLA transgenes to test this hypothesis. The data presented here describes the advantage RA-associated *0401 molecules provide over *0402 in clearing infection even though a difference in trafficking leads to binding self-antigens which may cause autoimmunity. The observations show that *0401 mice clear influenza virus infection and generate cross-reactive immunity while *0402 mice generate much higher antibodies than *0401 mice but do not generate cross-protective immunity. Moreover, we show that *0401 molecules traffic through the late endosome/lysosome while *0402 predominantly enters the early endosome. This difference in intracellular trafficking routes could result in differential antigen presentation and thus an increase in association with autoimmunity.

## Results

### *0401 mice generate cross-protective immunity to influenza strains

**Figure 1:**
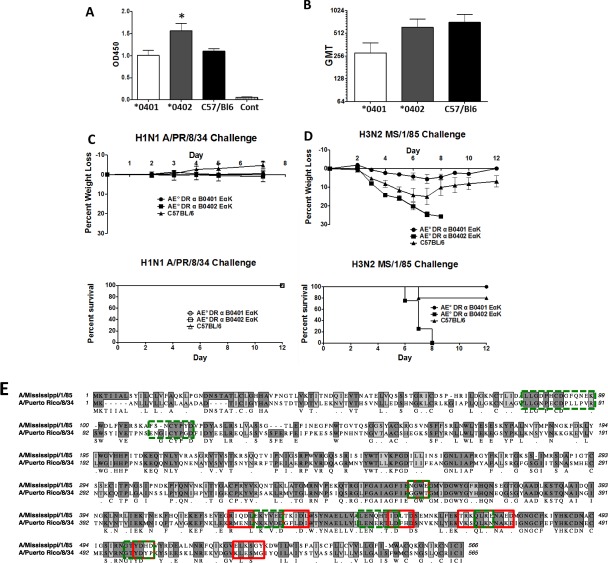
HLA-RB1*0401 leads to protection against heterologous influenza virus. Transgenic and control C57/BL6 mice were immunized with recombinant Ad5-ΔE1/3 virus expressing the full-length hemagglutinin of influenza A/PR/8/34 (N = 5 mice/group). Sera were analyzed for (**A**) total influenza binding antibodies, *0402 mice vs *0401, C57/BL6 mice, P < 0.05, and (**B**) protective functional antibodies. Data represent geometric Mean titer (GMT) with 95% confidence limits. Mice challenged with mouse-adapted A/PR/8/34 influenza virus were monitored for (**C**) weight loss, disease and survival. The mice were subsequently challenged with a heterologous mouse-adapted H3N2 A/Mississippi/1/85 influenza virus and monitored for (**D**) weight loss and survival. N = 5/strain. (**E**) A Clustal alignment of the HA proteins belonging to the homologous and heterologous influenza challenges is shown. Dark gray and light gray shadow boxes indicate sequence identity and similarity, respectively. The protein regions recognized by sera from the *0401 mice are indicated by red boxes and the regions recognized by the *0402 mice are indicated by the green boxes.

To define if autoimmune susceptibility alleles are much more effective at clearing infections, we used arthritis-susceptible *0401 mice and -resistant *0402 mice for clearing influenza *in vivo*. Mice were immunized with a recombinant adenovirus type expressing the hemagglutinin (HA) of influenza virus A/PR/8/34 and sera were assayed for the overall anti-influenza A/PR/8/34 antibodies and functional hemagglutination inhibition (HI) titers. The *0402-vaccinated mice generated significantly higher levels of anti-influenza responses (Fig. 1A) as well as functional HI antibody titers as compared to the *0401-vaccinated mice (Fig. 1B). When infected with H1N1 A/PR/8/34 influenza virus, all vaccinated mice, *0401 and *0402 as well as control C57/BL6 mice, cleared the infection (Fig. 1C) as tested by weight and survival. However, when these mice were challenged 3 months post H1N1 infection with heterologous H3N2 MS/1/85 influenza virus, the *0401 mice (N = 5) survived and regained weight within 10 days, while the *0402 mice (N = 5) lost 30% weight by day 8 and died (Fig. 1D). Among controls, 20% of mice died by day 8 although the survivors did not completely regain normal weight after challenge suggesting that *0401 retains superior efficacy in clearing infection via cross-protective immune response. These observations suggest that even though both strains of mice are protected from influenza, only *0401 mice can generate cross-protective immunity.

**Figure 2:**
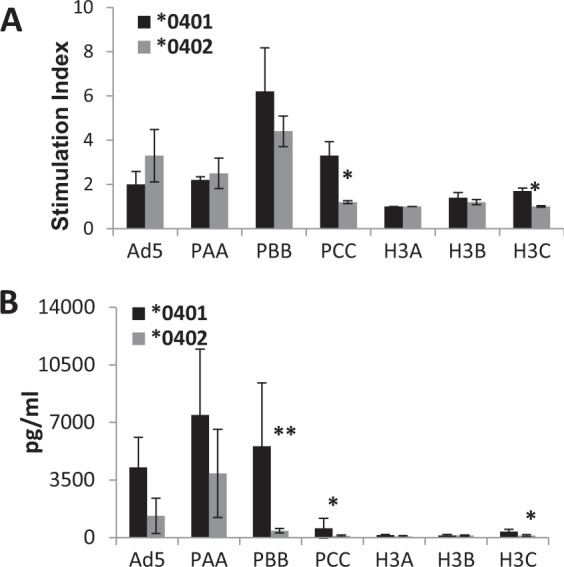
*0401 mice generate much robust response to H1N1 and H3N2 derived peptides. Overlapping peptides corresponding to the challenge strains H1N1 influenza virus A/PR/8/34 and a consensus H3 gene were used to stimulate splenocytes isolated the HLA transgenic mice. The peptides were combined into 3 pools (**A–C**). Pools A and B represent the more diverse globular head domain of the hemagglutinin (HA) protein and pool C represents the more conserved stalk domain. (**A**) The cell proliferation of splenocytes from mice vaccinated intramuscularly with 10^10^ vp/mouse of recombinant adenovirus type 5 E1/E3 deleted vector (Ad5ΔE1/E3) expressing the A/PR/8/34 HA gene is shown. (**B**) IFN-γ levels produced in the supernatants of the cultured cells in 2a. *P < 0.05, **P < 0.01.

To ascertain variation between antibodies generated in both strains for clearing infection, a peptide microarray of the MS/1/85 H3 protein was screened. Differences were observed in the peptide binding of antibodies in the vaccinated mice. The *0402 mice generated anti-head antibody responses against the MS/1/85 strain whereas the *0401 mice did not (Supplementary Fig. 1 and Fig. 2). Antibodies that recognize the globular head domain of influenza indicate strain specificity as this is the region of the HA that contains many of the neutralizing antibody epitopes. The *0402 mice generated antibody responses against the epitopes LLGDPHCDGFQNEK and FSNCYPYD while the *0401 mice did not (Fig. 1E). Both strains of mice generated anti-HA stalk directed antibodies. However, there was little overlap in the exact peptide sequences that were recognized by the sera from the vaccinated animals.

### *0401 mice generate more robust T cell response than *0402 mice post-vaccination

Splenocytes from naïve mice were stimulated with overlapping peptide pools from an H1N1 influenza HA gene and a consensus H3N2 influenza HA gene 2 weeks after vaccination. *0401 mice showed greater proliferation in response to the H1N1 peptides and H3C pool of H3N2 peptides than the *0402 mice (Fig. 2A). Supernatants of the *in vitro* cultures showed considerably greater IFN-γ production by the *0401 mice as compared to the *0402 mice (Fig. 2B). This may be indicative of a stronger role of T cell immunity in *0401 mice that resulted in greater levels of survival during the H3N2 influenza challenge. CD4+ T cells were sorted from vaccinated mice and cultured with matured bone marrow derived DCs also showed robust responses to H1N1 peptide pools PBB and PCC (Supplementary Fig. 3).

### Recycling trafficking of *0401 and *0402 molecules is through different compartments

**Figure 3:**
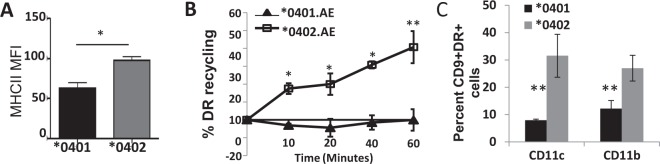
*0401 recycling is slower than *0402 (**A**) Flow cytometry-based expression of DR and (**B**) measurement of the surface recycling of MHCII molecules in BMDCs isolated from *0401 and *0402 mice; *P < 0.05, **P < 0.005. (**C**) Expression of CD9 and DR on dendritic cells expressing CD11c and CD11b, **P < 0.01, n = 3mice/group.

Based on the above observations and recent studies suggesting that endogenous processing of influenza is required to generate a robust CD4-dependent response^3^, we posited that trafficking of *0401 and *0402 molecules may be different. Initially we found that the surface expression of MHCII on *0401 bone marrow dendritic cells (BMDCs) was significantly lower than on *0402 cells (Fig. 3A, Supplementary Fig. 4). This data suggest that either there are differences in MHCII expression or, as we predict, there is a difference in receptor trafficking. Peptide uploading for nascent and recycling class II molecule occurs in distinct endosomal compartments. Recycling MHC II molecules upload peptides in early endosomes in DCs. Dissimilarity in receptor trafficking, if any, between *0401 and *0402 MHCII molecules was determined by using a flow cytometry directed recycling assay on *0401 and *0402 BMDCs (Fig. 3B, Supplementary Fig. 5). As shown, the *0402 MHCII allele exhibited a reduction in HLA-DR fluorescence and thus a steady increase in the percent of HLA-DR recycling over the time course of the assay. In contrast, the *0401 MHCII allele demonstrated a minimal reduction in HLA-DR fluorescence over the time course as indicative of defective or delayed recycling back to the cell surface. Taken together, these results suggest that these two alleles of MHCII have very distinct recycling properties, which could influence their ability to regulate adaptive immunity. Since CD9 has been shown to be involved in MHCII trafficking^12^, we analyzed co-expression of CD9 and DR on CD11c and CD11b cells (Fig. 3C). DCs from *0401 mice had lower co-expression of CD9 and DR suggesting they may be arrested in lysosomal associated membrane protein (LAMP) compartments.

**Figure 4:**
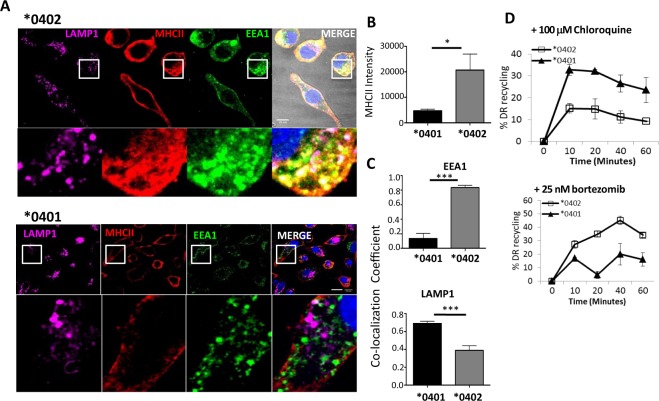
*0401 is localized to lysosomes and *0402 to early endosomes during recycling. (**A**) 3 D confocal microscopy image showing BMDCs from *0401 and *0402 mice stained for DR (MHCII), early endosomal antigen (EEA1) and lysosomal associated membrane protein 1 (LAMP1). (**B**) Quantification of MHCII integrated intensity from images in (**A**) using FIJI (NIH). (**C**) Images were analyzed for MHCII co-localization with (top) EEA1 and (bottom) LAMP1 using Pearson’s co-localization coefficient in ZEN (Carl Zeiss). Zoomed images are demarcated by white boxes. For each condition, >20 individual cells were imaged. Scale bars, 10 µm. Data represent mean ± SEM (n = 3 mice per group). MHCII recycling in BMDCs treated with (**D**) lysosome inhibitor, Chloroquine, and (**E**) protease inhibitor, Bortezomib. Data represent mean ± SEM (n = 3 mice per group) from two independent experiments, P < 0.05.

Next we examined the localization of surface-derived MHCII after endocytosis by an immunofluorescence-directed endocytosis assay. BMDCs from transgenic mice were cultured with anti-DR antibodies and internalization of the DR molecules was visualized by confocal microscopy (Fig. 4A). Single plain images from z-stacks of BMDCs from *0402 mice displayed higher HLA-DR than from *0401 mice, confirmed by significantly higher integrated intensity (P < 0.05) (Fig. 4B). BMDCs from *0401 mice showed ~4-fold less HLA-DR fluorescence intensity than *0402 BMDCs. *0401 BMDCs also showed a ~50% lower co-localization of HLA-DR with early endosomal antigen (EEA1) and a ~40% increase in co-localization with LAMP1^+^ compared to *0402 (Fig. 4C). On the other hand, *0402 BMDCs showed a 4-fold increase in MHCII co-localization with EEA1 compared to *0401 BMDCs (P < 0.001). The data suggest that these MHCII molecules utilize different endosomal trafficking routes and MHCII in *0401 may be sequestered more often to lysosomes for degradation leading to the loss of recycling and lower surface expression.

Since *0401 co-localized with LAMP1, we next determined if *0401 molecules traffic through lysosomes. BMDCs were cultured with the lysosomal inhibitor, Chloroquine, and protease inhibitor Bortezomib^13,14^ and trafficking was analyzed using the recycling assay. Chloroquine and Bortezomib restored the expression and recycling of *0401 molecule confirming the difference in trafficking of the 2 molecules (Fig. 4D,E, Supplementary Figs. 6 and 7). This data suggest that the lower MHCII expression of *0401 compared to *0402 is due to increased trafficking of MHCII to the lysosome for degradation in *0401 cells.

### *0401 mice generate a robust response to non-TCR and TCR-dependent antigens

**Figure 5:**
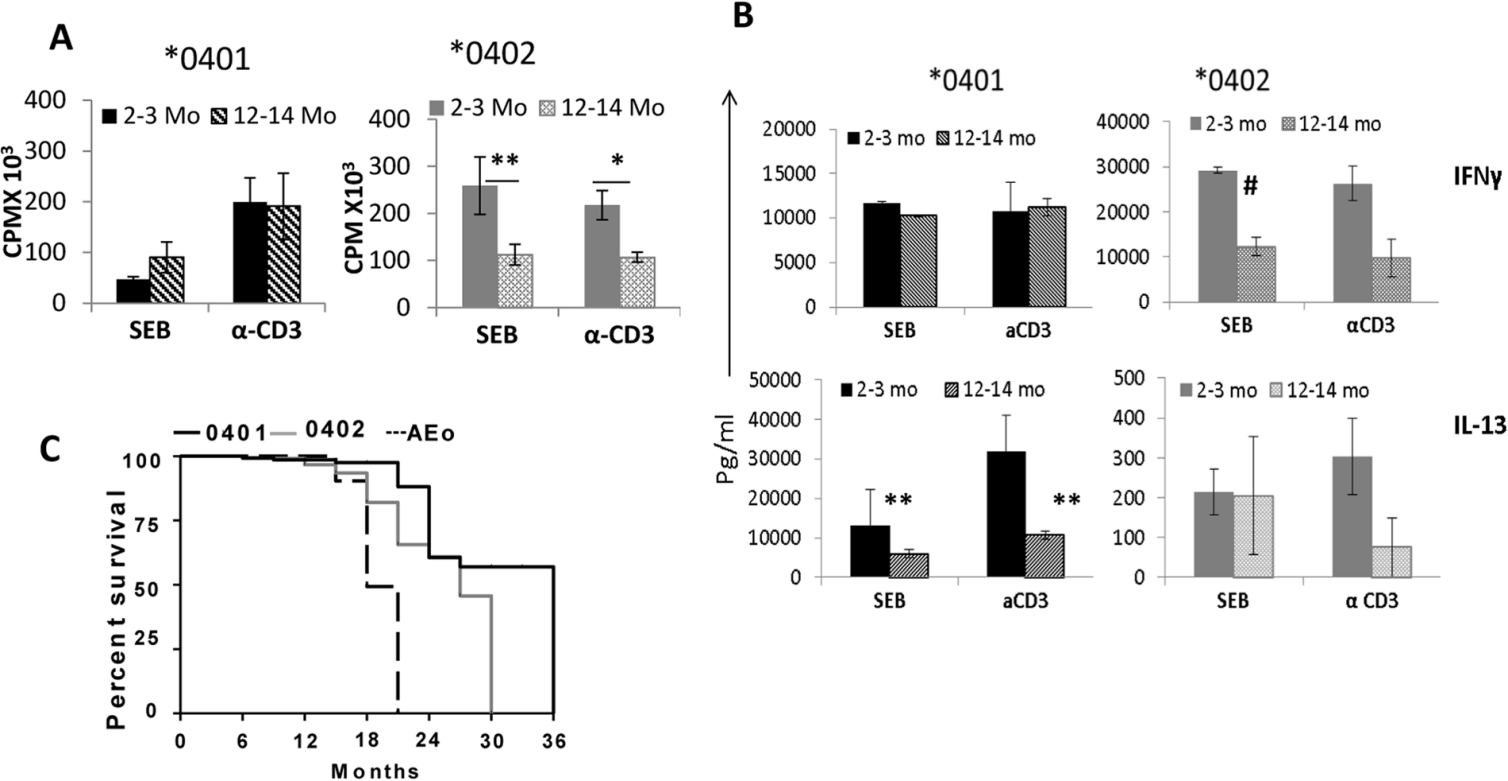
HLA-DRB1*0401 mice survive longer than *0402 mice and generate differential immune response to superantigen. (**A**) *In vitro* response to Staphylococcus enterotoxin B (SEB) and α-CD3 of splenocytes harvested from naïve mice at indicated time points, *P = 0.06, **P = 0.03 and (**B**) cytokine response from the culture supernatants from 4A. ^#^P = 0.01, **P = 0.03. Data represents Mean ± SEM (N = 3 mice/strain). *0401 vs *0402, IFN-γ, 2-3mo- SEB, *P < 0.001, CD3, P < 0.07; IL-13, *0401 vs *0402, SEB and CD3, for 2-3mo and 12-14mo P < 0.001. (**C**) Kaplan Meier plot for *0401 (N = 23), *0402 (N = 21) and AEo (N = 10) naïve mice, *0401 vs *0402, P < 0.001.

The observations with influenza suggested that, as per hypothesis, they should generate a robust response to pathogens. We have previously shown that *0401 mice generate robust TLR4 and TLR9 –dependent responses while *0402 mice do not^8,10^. Since influenza is much more severe in older individuals, we tested if there is an age-dependent difference in response to Staphylococcus enterotoxin B (SEB), a superantigen, and a TCR-dependent response between the 2 strains. Interestingly, an opposite trend was observed in both strains, *0402 mice generated a much more robust response in young mice only while *0401 mice generated a similar response in young and old mice (Fig. 5A). Also, the immune response profile was different between the strains with *0401 mice producing very high levels of IL-13, while *0402 mice produced much higher levels of IFN-γ (Fig. 5B). On the other hand, IL-6 was produced similar in both strains (Supplementary Fig. 8). These data suggest that even though both strains generate response to superantigens, signaling pathways by which they clear infection are different.

### *0401 mice have a longer life span than *0402 mice

**Figure 6:**
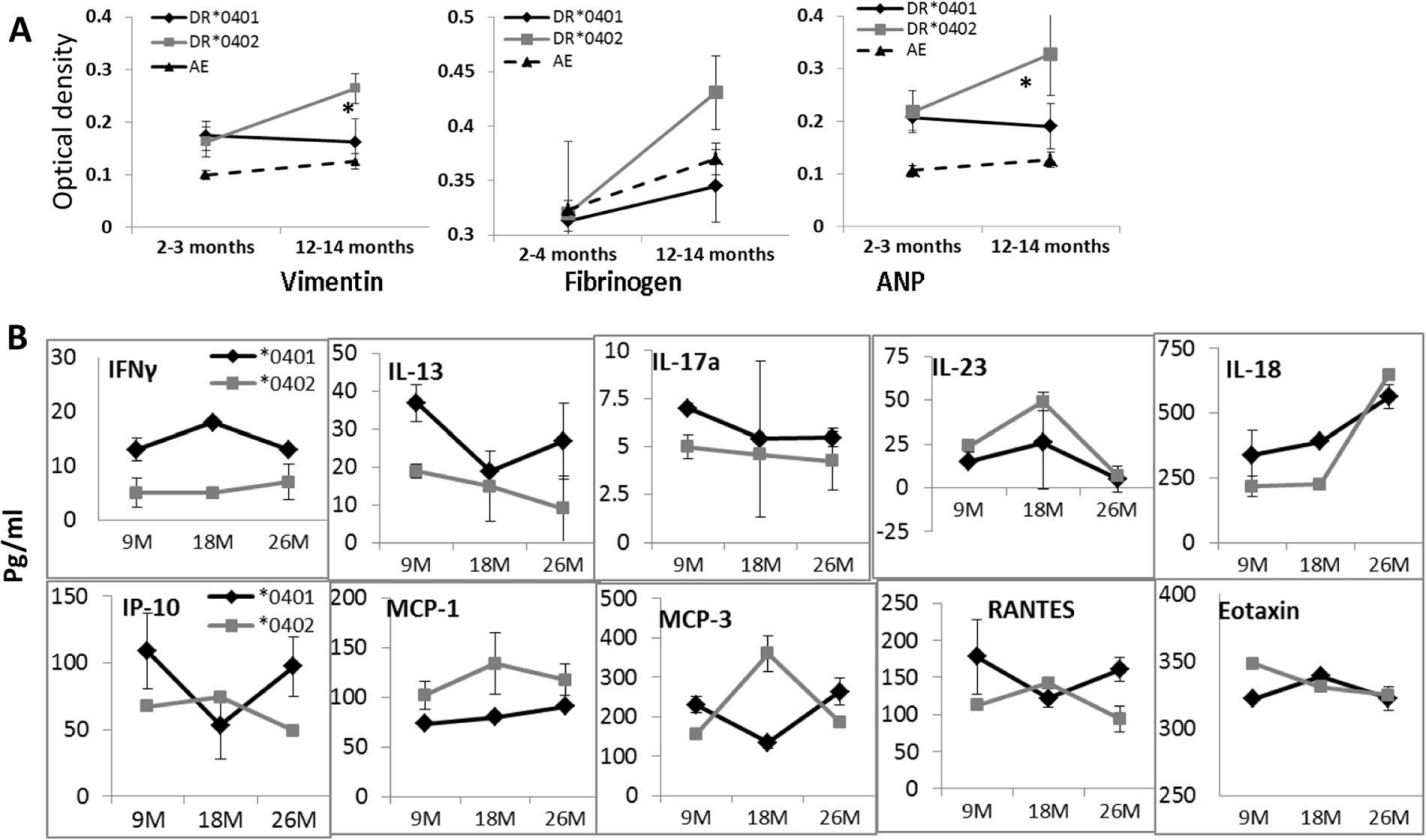
Aging mice are characterized by differential antibodies and cytokine response. (**A**) Sera from mice at indicated times was tested for antibodies to Vimentin, Fibrinogen and atrial natriuretic peptide (ANP); Vimentin and ANP, *0402 young vs old P < 0.05, ANP old *0401 vs *0402, P < 0.02, Vimentin, old *0401 vs *0402, P < 0.08. Control young AEo vs *0401 and *0402 mice for Vimentin and ANP, P < 0.02 and old AEo vs *0402 for Vimentin and ANP, P < 0.01 (N = 4–6/group). Sera cytokines at indicated ages in naive mice (N = 5–6/strain/time point). *0401 vs *0402, *P < 0.04, **P < 0.005, ^#^P < 0.05.

To determine if *0401 provides an evolutionary advantage; we tested the antagonistic pleiotropic effect of the *0401 and *0402 genes by assessing the length of survival of naïve mice under normal conditions. We have previously published that *0401 mice develop collagen-induced arthritis while *0402 mice don’t. We assumed that *0402 mice are generally healthy and should live longer. As shown in Fig. 5C, *0401 mice had a longer life span as compared to *0402 mice (P < 0.001). An immune response to self-proteins has been linked to autoimmunity, cancers and other diseases^15–18^, that can cause mortality at a young age. To rule out these factors as cause for the shorter life span of *0402 mice, we tested auto-reactivity to some self-antigens including Vimentin, fibrinogen and atrial natriuretic peptide (ANP), by measuring antibody production to these proteins in sera of young and aged naive mice. Interestingly, both strains showed similar levels of antibodies at a young age though antibodies to self-antigens, Vimentin and ANP, were significantly higher in aged *0402 mice, P < 0.05 (Fig. 6A) even though *0402 mice are not susceptible to autoimmunity. Interestingly, *0401 mice were characterized by higher proinflammatory cytokines, IFN-γ, IL-17, IL-18, IL-13 and chemokines IP-10 and RANTES (CCL5) levels, while *0402 mice had higher levels of IL-10 and MCP-3 and MCP-1 levels, suggesting a different immune profile in naïve mice of both strains (Fig. 6B). Among all the cytokines and chemokines, the significant differences throughout the life were observed for cytokines, IFN-γ and MCP-1. These data suggest that in *0401 mice, high CCL5 leads to maturation of DCs and activation of IFN-γ producing T cells while CCL2 leads to overall Th2 response in *0402 mice^19^.

## Discussion

Vaccines are given routinely to protect individuals from various infections. However, vaccines do not provide similar protection to all individuals. We hypothesized that even though vaccines are promiscuous in nature, genetic factors like HLA alleles, determining cytokine profile in response to infection and vaccination, regulate the final outcome. Evolutionarily, certain HLA alleles are superior in providing protection by presenting multiple epitopes for the activation of T cells. Most autoimmune diseases have been associated with the presence of certain HLA class II molecules. Approximately 30% of Caucasians carry DRB1*04, an allele with more than 50 subtypes. Of all the DRB1*04 alleles, *0401 is the most frequent allele occurring with a frequency of 57% as compared to *0402 which occurs with a frequency of 6.3%^20^. However, *0401 has been associated with various autoimmune diseases including RA^4^. Current observations suggest that even though both strains of mice are protected from influenza, only *0401 mice can generate cross-protective immunity. One can speculate that an activation of these cells post infection may lead to autoreactivity. This is supported by previous observations that *0401 mice harbor memory Th17 CD4+ T cells to self-epitopes^10^. Viral infection in genetically susceptible mice has been shown to lead to autoimmune disease^21^. DRB1*0402 mice, on the other hand, while able to clear an infection, succumbed to challenge with heterologous infection suggesting a defective T cell memory since both strains generated response to all epitopes except two. Influenza vaccine has been shown to induce heterologous protective immunity in pigs also^22^. *0401 mice showed greater proliferation responses and strikingly higher IFN-γ responses as compared to the *0402 mice. Considering that the only difference between these strains is the HLA allele, it is a clear indication that both class I and class II alleles play a significant role in the development of immunity to pathogens and, most likely, autoimmunity. The interconnections between the arms of the immune responses are far from being well understood, however, these data may provide some insights.

Recycling MHC II molecules upload peptides in early endosomes in DCs. A recent study showed that endogenous processing is required for an efficient CD4 primary response to influenza^3^. Based on our observations showing *0401 and *0402 localization in different compartments while recycling, one can speculate that they have access to different peptides. Since lysosomes can fuse with auto-phagosome and contain cytosolic proteins as well as self-peptides from autophagy, availability of self-peptides to be presented by class II molecules is high. In support of this, gliadin, an antigen for celiac disease, has been shown to translocate to lysosomes before presentation to T cells^23^. Indeed, naïve *0401 mice harbor DR-restricted CD4+ T cells reactive to type II collagen peptide which are Th17^10^. Also, naïve CD4+ T cells from *0401 mice can generate response to Vimentin, a major cytoskeletal protein which is released in apoptotic cells^24^. The early and late endosomes have been suggested to generate different MHC II-peptides from an antigen^6,25^. While late endosome and lysosome uploading of peptides to MHC II is DM-dependent, epitope uploading in early endosomes is not. Peptide elution from *0401 and *0402 showed Vimentin-derived epitopes from both recycling molecules (unpublished data), some specific to each molecule while other were similar. Interestingly, lower numbers of peptides were eluted from *0401 molecules as compared to *0402 molecules. This difference between the 2 molecules may lead to different peptides being loaded and presented from pathogens as well as self-proteins and, in part, explain the shared epitope association with RA. However, different binding capability of the 2 molecules may explain the differences in the eluted peptides. Future work on the eluted peptides will help determine their pathogenicity.

Non-antigen specific immune response also showed different signaling pathways for the 2 molecules. In response to superantigens, as *0401 mice produced higher levels of IL-13 while *0402 mice produced IFN-γ. This data supports our previous observations that cells from *0402 mice can be driven to produce higher IFN-γ than *0401 mice^10^. An increased production of IL-13 has been shown in patients with RA as well arthritic humanized *0401 mice^26,27^. IL-13 has pleiotropic effects and differentiates B cells into antibody producing cells^28,29^ and contributes to DC growth from progenitors and induction of chemokine CCL18 (47, 48). IFN-γ can enhance production of chemokines like RANTES and MCP-1^30,31^, as observed in *0402 mice, suggesting IFN-γ induced production of chemokines may be an initial response for protection from pathogens.

The observations reported here delineate how MHC II molecules may regulate the outcome of infections. The data further sheds light on the evolutionary advantage of certain MHCII molecules even though they are associated with susceptibility to diseases. This antagonistic pleiotropic effect has an advantage of enhanced longevity and explains evolutionary selection of MHCII genes. The data suggest a need to further test other MHC II alleles associated with autoimmune diseases to prove if recycling differences predict the response to infections and vaccinations for certain individuals.

## Methods

### Transgenic mice

The generation of HLA-DRB1*0401.AEo and *0402.AEo transgenic mice has been described previously^8,32,33^. Mice of both sexes (8–104 weeks of age) used in this study were bred and maintained in the pathogen-free Colony at the Mayo Clinic, Rochester. All the experiments included littermate controls, AEo and were carried out with the approval of the Mayo Clinic Institutional Animal use and care committee (IACUC) using ARRIVE guidelines.

For aging studies, mice were kept in conventional colony and monitored for any obvious signs of distress. Sera were collected from naïve mice at various times, ages 2–3 months (young), and 12–14 month (old), and also at 9month, 18 month and 26 month.

### Infections and vaccination

Transgenic mice and control C57/BL6 mice were vaccinated with a recombinant adenovirus type 5 E1/E3 deleted vector expressing the hemagglutinin (HA) of H1N1 influenza virus A/PR/8/34. Intramuscular immunizations consisted of a 50 µl vaccine(10^10^ vp/mouse) diluted in PBS and injected using a 27 gauge needle into both quadriceps in two 25 µl injections. Three weeks post-vaccination the mice were bled and challenged with 10 µl of a lethal mouse-adapted homologous influenza A/PR/8/34 virus. Briefly, anesthetized mice were challenged intranasally with 100MLD_50_ of mouse-adapted influenza A/PR/8/34 virus into each nare. Baseline weight was determined at the time of the challenge and the mice were monitored for weight loss and disease. For infection, 10 µl of influenza was instilled into the nares of the mouse (10 µl/nare). Three months post-challenge, they were challenged with 1.0 MLD_50_ of heterologous MS/1/85 mouse-adapted influenza virus. All influenza viruses were obtained from ATCC or the Biodefense and Emerging Infectious Diseases Repository under the USDA.

### ELISA and hemagglutination inhibition (HI) titers

Sera from the vaccinated mice were screened for anti-influenza A/PR/8/34 antibodies as previously described^34^. Briefly, A/PR/8/34 virus harvested from the chorioallantoic fluid of infected embryonated eggs was dialyzed against 3–1 L changes of PBS overnight using a Spectra/Por Float-A-Lyzer (MWCO: 1000 KD). This virus was used to coat the ELISA plates at 4 °C overnight. The plates were blocked using 3% BSA and then incubated with the diluted sera from vaccinated mice. The mouse anti-Influenza antibodies were detected using Pierce Goat Anti-mouse (H + L)-HRP. The ELISA was developed using 1-Step Ultra TMB-ELISA, stopped with sulfuric acid and the OD450 was read using an 880DTX multimode microplate reader. The HI titers were determined as previously described^35^. Briefly, sera were diluted two-fold in 50 µl of DPBS in a 96-well round bottom plate and incubated with four hemagglutination units (HAU) of A/PR/8/34 virus (diluted into 50 µl) at room temperature (RT) for 1 hr. 50 µl of a 1% chicken RBC solution was added to the sera-virus dilutions and incubated at RT for 1 hr. The HI titer was measured as the reciprocal of the highest serum dilution to inhibit hemagglutination.

Antibodies to Vim, Fibrinogen and ANP in sera from 2–3 month and 12–14 month old naïve mice were measured by standard ELISA using plates were coated with the respective antigens.

### Cytokines and T cell proliferation

Cytokines from sera collected at various time points were measured using the Bio-Plex array system with the mouse cytokine 23-plex panel as per manufacturer’s instructions and analyzed with Bio-Plex manager 2.0 software (Bio-Rad laboratories, Hercules, CA).

Splenocytes (1 × 10^6^) harvested from mice vaccinated with 10^10^ vp/mouse of Ad5ΔE1/E3 expressing the A/PR/8/34 HA gene for 2 weeks were challenged *in vitro* with overlapping peptide pools from an H1N1 influenza HA gene and a consensus H3N2 influenza HA gene. The peptides were combined into 3 pools A, B and C. Pools A and B represent the more diverse globular head domain of the HA protein and pool C represents the more conserved stalk domain. Supernatants were used to measure IFN-γ by ELISA.

In another experiment, bone marrow cells harvested from transgenic mice were cultured for 5 days in medium (RPMI1640 containing 1% penicillin-streptomycin and supplements) with GM-CSF (10 ng/ml) and IL-4 (1 ng/ml) for maturation of dendritic cells (DCs) as described previously^10^. After 5 day culture, mature CD11C + DC were cultured with splenic CD4 T cells (1 × 10^6^ cells) isolated by positive selection (Miltenyi Biotech) and T cell proliferation was measured.

*In vitro* response to LPS and CPG (10ug/ml) was studied by challenging splenic cells harvested from naïve mice.

### Peptide microarray

The sera from the vaccinated mice were screened against 15-mer peptides overlapping peptides representing the full-length of A/Mississippi/1/85 HA protein to determine the specific linear antibody epitopes induced by the Ad-vectored HA vaccine. The PEPperCHIP® Peptide Microarrays were produced by B-Bridge International (Cupertino, CA) using a Poly(ethylene glycol)-based graft copolymer coating with a thickness of 13,5 nm and an additional three amino acid linker (ß-alanine, aspartic acid, ß-alanine) to each peptide.

The microarrays were probed using sera from immunized transgenic mice as described in the Pepper CHIP immunoassay protocol^36^. Briefly, the microarray was blocked with PBS (pH 7.4/0.05% Tween 20/1% BSA) for 30 min. at room temperature, then washed with standard buffer (PBS, pH 7.4/0.05% Tween 20) and incubated with sera diluted 1:1000 in standard buffer +0.1% BSA for 1 hour. Cy3 Goat anti-mouse IgG (H + L) antibody (Life Technologies, Eugene, OR) was used for detection of the specificity of the bound antibody. The array was washed and dried for scanning. Control peptides were detected using standard anti-HA and anti-FLAG DyLight 680 and 800 antibodies as previously described^36^. The microarrays were scanned at 5 µM resolution using the Axon GenePix 4000B scanner.

### Immunofluorescence and expression of DR

BMDCs were cultured on #1.5 LabTek II eight-chambered coverslips (Nunc) for at least 30 minutes to allow firm adhesion. Cells were fixed by addition of ice-cold fixative (4% paraformaldehyde and 0.5% glutaraldehyde in PBS) and incubated for 30 min at room temperature in the dark, followed by permeabilization with 0.2% Triton X-100 in PBS for 15 min. Cells were then cultured with 10 µg/ml primary antibodies (Abs) in IF buffer (TBS plus human serum cocktail) overnight at 4 °C. Primary Abs consist of a mouse monoclonal anti-HLA-DR (L227), rat polyclonal anti-LAMP1 (BD Biosciences), and goat polyclonal anti-EEA1 (Santa Cruz) diluted in IF buffer. Cells were washed in PBS and incubated with secondary Abs (1:500 dilution in imaging buffer) for 1 hr. at room temperature. After washes with PBS and addition of Hoechst 33342 nuclear stain, Slow Fade Gold antifade reagent (Molecular Probes) was added to the wells. Images were obtained with an LSM-710 laser scanning confocal microscope with a 100X/1.4 Oil Plan-Aprochromat objective lens using ZEN software (Carl Zeiss). Each image represents an individual slice taken from a z-stack comprised of several slices at 0.25 µm depth.

BMDCs from mice were suspended at 10^6^ cells/ml in FACS buffer and stained with anti-HLA-DR-AF647 and analyzed using a flow cytometry. Data were analyzed using FlowJo 8.8.7 software (Tree Star Ashland, OR). Expression of DR and CD9 was analyzed by FACs on CD11c and CD11b cells using conjugated antibodies.

### MHCII endocytosis assay and recycling assay

To specifically track the localization of surface-derived MHCII after endocytosis, we performed an IF directed endocytosis (internalization) assay as described previously^37^. BMDCs were cultured on #1.5 LabTek II eight-chambered coverslips as described above. After 30 min culture, the cell media was replaced with serum-free DMEM containing 10 µg/ml anti-HLA-DR and then incubated at 37 °C for 30 min to allow internalization.

For recycling assay, BMDCs from mice were surfaced labeled with an anti-HLA-DR-AF647 antibody for 30 min, washed in DC culture media (RPMI-10% FBS + GM-CSF + IL-4), and incubated for 30 min to allow internalization. Cells were spun down and re-suspended in FACS buffer stripping solution (PBS containing 2% BSA Fraction V [Sigma Aldrich] and 0.1% NaN_3_, pH 2.5) for 10 min on ice. Cells were then washed in FACS buffer (pH 7.4 PBS containing 2% BSA Fraction V [Sigma Aldrich] and 0.1% NaN_3_) and re-suspended in DC culture media and incubated for 0, 10, 20, 40, and 60 min to allow resurfacing of the internalized HLA-DR. Following incubation, cells were spun down and re-suspended in FACS buffer stripping solution for 10 min on ice. Cells were then washed, re-suspended in 500 µl FACS buffer and analyzed by flow cytometry using a FACSCanto II flow cytometer (BD Biosciences). Data were analyzed using FlowJo 8.8.7 software. The percentage of recycled HLA-DR was measured using the equation (T_0_ − T_x_)/T_0_ × 100. T_0_ represents the mean fluorescence of cells following the second acid strip at time zero and T_x_ is the mean fluorescence intensity of cells stripped at each time point. The acid stripping method was adapted from previous publication^37^. The recycling assay measures the trafficking of intracellular/endocytosed MHCII receptors bound to a fluorescently conjugated anti-DR antibody.

### Image analysis

Co-localization was assessed by Pearson’s correlation coefficient and overlap coefficient using ZEN software (Carl Zeiss). Integrated intensity measurements were performed using Fiji: Image J (National Institutes of Health: http;//rsb.info.nih.gov/ij/).

### Statistical analysis

Data are expressed throughout as mean ± standard error mean (SEM). Data sets were compared using the two-tailed unpaired Student’s t-test. For multiple groups, one-way ANOVA repeated measurements plus Bonferroni’s Multiple Comparison Test was used. Statistical analysis (Student’s t*-*test, ANOVAs, and column statistics) and graphing were performed using Prism 4 (GraphPad, Lo Jolla, CA). Comparison of survival curves was done by Kaplan Meier survival curve. Differences were considered statistically significant when *p* < 0.05.

## Supplementary information

Supplementary information 
