# Supplementary material for: Immunity to Influenza is dependent on MHC II polymorphism: study with 2 HLA transgenic strains
Source: Sci Rep. 2019 Dec 13;9:19061. doi: 10.1038/s41598-019-55503-1 (PMC6911063; doi:10.1038/s41598-019-55503-1)
Supplement: Supplementary file 1 — Supplementary information [file 41598_2019_55503_MOESM1_ESM.pdf]

Immunity to Influenza is dependent on MHC II polymorphism: study with 2 HLA transgenic strains

David Luckey[1], Eric Weaver [1#], Douglas G Osborne[1†], Daniel D Billadeau[1] and Veena Taneja[1*]

1 Department of Immunology Mayo Clinic, Rochester, MN

***Corresponding author**
Veena Taneja
Department of Immunology
Mayo Clinic,
200 First Street, SW
Rochester, MN 55905
Email: taneja.veena@mayo.edu
Ph: 507 284 4541

Present address

School of Biological Sciences, Nebraska Center for Virology, University of Nebraska,

Lincoln, NE† Department of Dermatology, University of Colorado, Aurora, CO

Supplementary Figure1

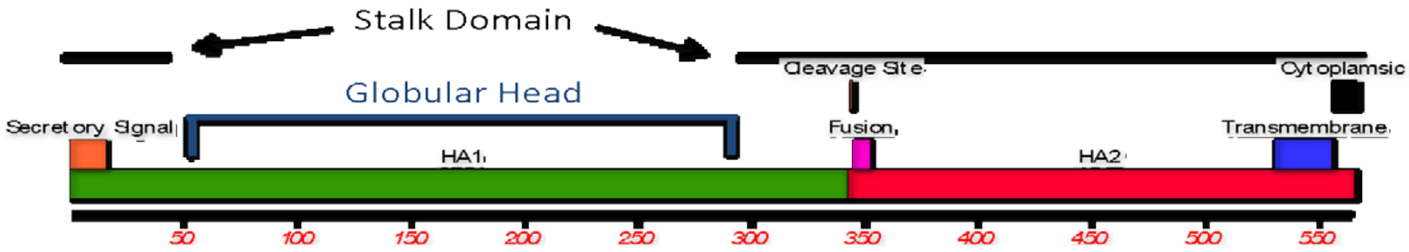

Recombinant Ad5-ΔE1/3 virus expressing the full-length hemagglutinin of influenza A/PR/8/34

Supplementary Figure 2

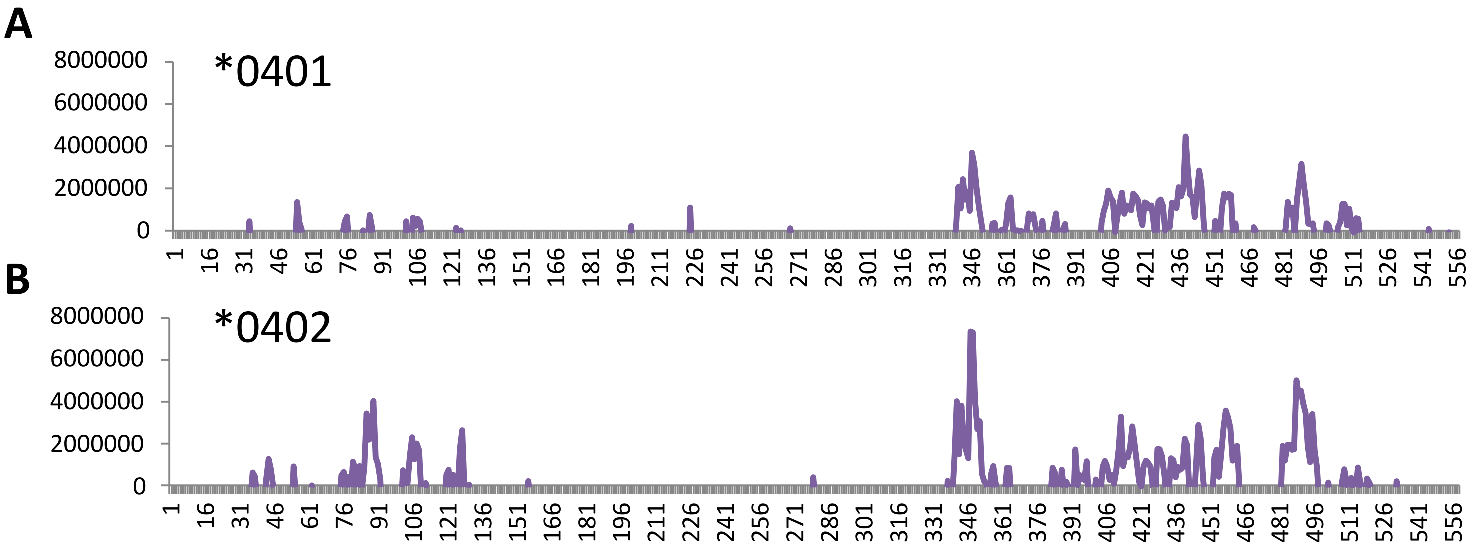

Pooled sera, collected 3 weeks after vaccination, from HLA-transgenic mice were used to measure the anti-H3 influenza antibodies. Antibodies responses against the MS/85 peptides are indicated by the blue lines and indicate the presence of linear B-cell epitopes. The linear antibody epitopes recognized by *A)* DRB1*0401 and B) DRB1-*0402 mice, N=5/group.

Supplementary Figure 3

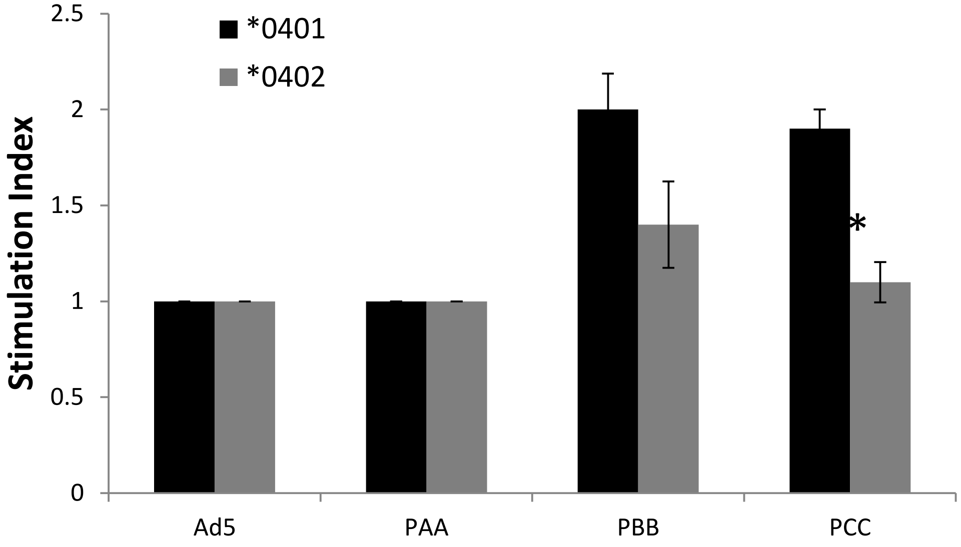

Bone marrow cells harvested from transgenic mice were cultured for 5 days in medium (RPMI1640 containing 1% penicillin-streptomycin and supplements) with GM-CSF (10ng/ml) and IL-4 (1 ng/ml) for maturation of dendritic cells (DCs). After 5 day culture, mature CD11C+ DC were cultured with splenic CD4 T cells isolated by positive selection (Miltenyi Biotech) and T cell proliferation was measured, *P<0.01 Peptides for H3N2 were not tested.

Supplementary Figure 4

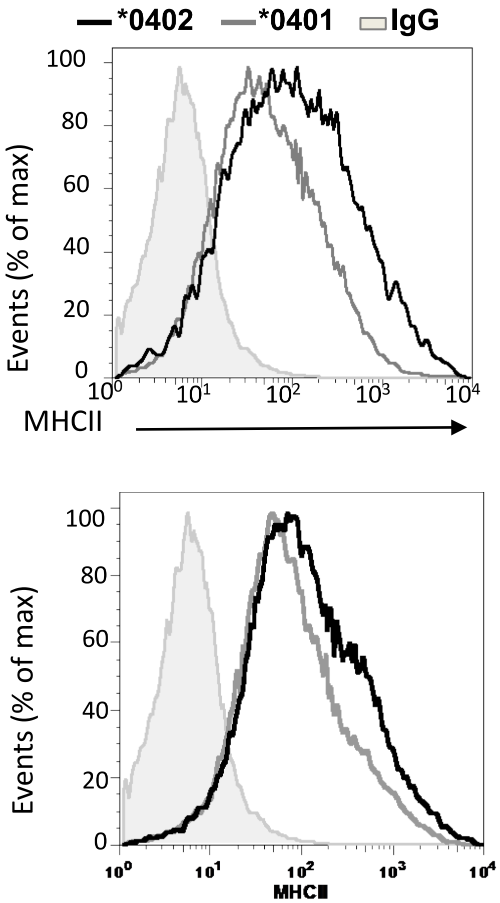

Flow cytometry-based expression of DR in BMDCs in *0401 and *0402 mice. Cells were cultured with conjugated anti-DR antibodies (L227) and expression analyzed by Flow cytometry. IgG was used as isotype control. Set of 2 experiments is shown

Supplementary Figure 5

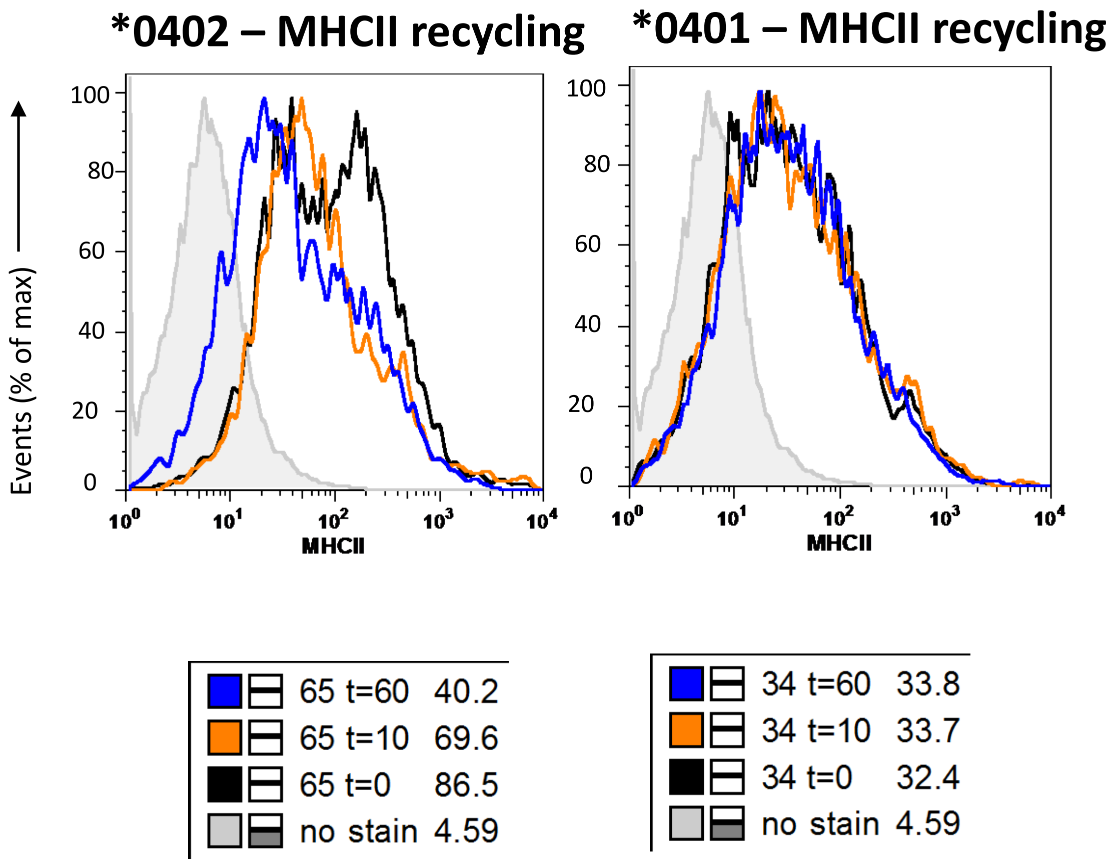

Measurement of the surface recycling of MHCII molecules in BMDCs isolated from *0401 and *0402 mice. Cells were cultured with conjugated anti-DR antibodies (L227) and expression analyzed by Flow cytometry. Peak MFI provided. IgG was used as isotype control. Acid stripping method was used to determine recycling

Supplementary Figure 6

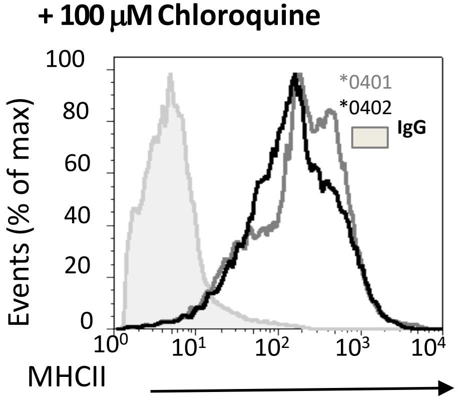

MHCII recycling in BMDCs treated with lysosome inhibitor, Chloroquine. BMDCs were cultured in the presence of Chloroquine and acid stripping method was used for recycling.

Supplementary Figure 7

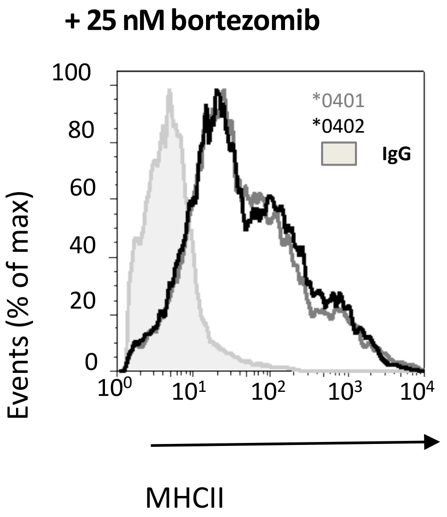

BMDCs were cultured in presence of Bortezomib and MHCII recycling was determined by FACS using acid stripping method and staining with conjugated L227 (anti-DR antibodies).

Supplementary Figure 8

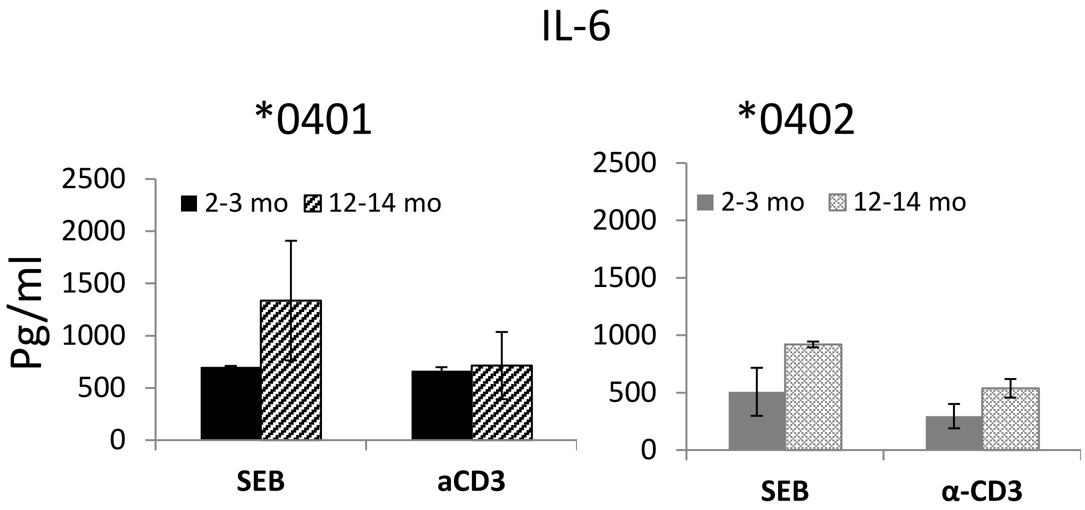

T cell response to TCR independent (Staphylococcus enterotoxin B (SEB) and TCR-dependent (α-CD3) (N=3 mice/strain/time point) of splenocytes harvested from naïve mice at indicated time points and IL-6 measured in the supernatant.
